# From gut to placenta: understanding how the maternal microbiome models life-long conditions

**DOI:** 10.3389/fendo.2023.1304727

**Published:** 2023-12-15

**Authors:** Jonathan Ruiz-Triviño, Daniel Álvarez, Ángela P. Cadavid J., Angela M. Alvarez

**Affiliations:** ^1^ Grupo Reproducción, Departamento de Microbiología y Parasitología, Facultad de Medicina, Universidad de Antioquia - UdeA, Medellín, Colombia; ^2^ Semillero de Investigación en Alteraciones de la Gestación y Autoinmunidad (SIAGA), Universidad de Antioquia - UdeA, Medellín, Colombia; ^3^ Grupo de Investigación en Trombosis, Facultad de Medicina, Universidad de Antioquia - UdeA, Medellín, Colombia; ^4^ Departamento de Obstetricia y Ginecología, Facultad de Medicina, Universidad de Antioquia - UdeA, Medellín, Colombia

**Keywords:** microbiota, dysbiosis, pregnancy, microbial metabolites, epigenome, fetal development

## Abstract

The microbiome -defined as the microbiota (bacteria, archaea, lower and higher eukaryotes), their genomes, and the surrounding environmental conditions- has a well-described range of physiological functions. Thus, an imbalance of the microbiota composition -dysbiosis- has been associated with pregnancy complications or adverse fetal outcomes. Although there is controversy about the existence or absence of a microbiome in the placenta and fetus during healthy pregnancy, it is known that gut microbiota can produce bioactive metabolites that can enter the maternal circulation and may be actively or passively transferred through the placenta. Furthermore, the evidence suggests that such metabolites have some effect on the fetus. Since the microbiome can influence the epigenome, and modifications of the epigenome could be responsible for fetal programming, it can be experimentally supported that the maternal microbiome and its metabolites could be involved in fetal programming. The developmental origin of health and disease (DOHaD) approach looks to understand how exposure to environmental factors during periods of high plasticity in the early stages of life (e.g., gestational period) influences the program for disease risk in the progeny. Therefore, according to the DOHaD approach, the influence of maternal microbiota in disease development must be explored. Here, we described some of the diseases of adulthood that could be related to alterations in the maternal microbiota. In summary, this review aims to highlight the influence of maternal microbiota on both fetal development and postnatal life, suggesting that dysbiosis on this microbiota could be related to adulthood morbidity.

## 1 Introduction

The microbiome comprises a community of microorganisms that inhabit a body space or environment, as well as their genomes and surrounding environmental conditions (1). Some of the functions attributed to microbiota are promoting the development of the immune and central nervous systems, protecting against the invasion of pathogens in the body’s mucous membranes, modulating metabolic processes, and synthesizing active compounds, among others (2, 3).

There is substantial controversy regarding the presence of microorganisms in prenatal intrauterine locations and the consequent environment of sterility in which the fetus develops: the traditional dogma that the human fetal environment is sterile and that the neonate microbiome is acquired during and after birth (4–11) stands against the evidence supporting that the healthy human placenta harbors a unique low-biomass microbiome composed of non-pathogenic commensal microbiota (12–14); although recent studies indicate that the detected microbial signals are likely the result of contamination during sample collection or during DNA extraction and sequencing (15–17). That controversy about the existence or absence of a microbiome in the placenta and fetus of a healthy pregnancy is not the focus of this review. We invite you to check out the recent papers on the subject (18–20).

In contrast, relevant to the scope of this article, current evidence supports that microbiota-derived metabolites or pathogenic microorganisms may be transferred through the placenta during fetal development, which has adverse outcomes for the fetus. Dysbiosis in the gut microbiota (GM) and dysfunction in the integrity of the gut barrier have been associated with multiple diseases. In such cases, the microbial communities and derived metabolites can enter the extraintestinal tissues or the host circulation, exert physiological effects, and activate several signaling pathways, contributing to disease development (21–23).

The microbiome is a new factor in the developmental origin of health and disease (DOHaD). Since microbiota, pathogenic microorganisms, and their derived metabolites influence the epigenome, exploring the evidence about epigenetic mechanisms activated in response to maternal microbiota alterations is gaining relevance. Once it has been understood that the microbiome (and its effects on the metabolome) can influence fetal programming, scenarios can be addressed in which it is possible to associate changes in the maternal microbiome and the development of disease in offspring. In summary, this review aims to highlight the influence of maternal microbiota on fetal development and postnatal life, which can lead to an understanding of how dysbiosis on maternal microbiota can be related to adulthood morbidity.

## 2 Microbiome alterations in pregnancy disorders

The exact contribution of the maternal microbiome to pregnancy complications or adverse fetal outcomes is not entirely understood. It is widely accepted that some gestational disorders are involved with fetal complications (24, 25), fetal programming, and the origin of adult chronic noncommunicable diseases, in as much as changes in the intrauterine environment can affect the metabolic regulation and development of specific tissues, such as adipose tissue and the cardiovascular system (26, 27). Setting aside the discussion about the presence or absence of the placental microbiome in the context of healthy pregnancy, studies have reported changes in the GM profile in pregnant women with gestational disorders such as preeclampsia (PE), gestational diabetes mellitus (GDM), fetal growth restriction (FGR), maternal obesity, among others. **Supplementary Table 1** describes the profile of the microbiota in such gestational alterations (28–43).

Besides, increased levels of plasma lipopolysaccharide (LPS) in PE patients were found, and recently the gut microbiota associated with the LPS synthesis in combination with increased placental LPS levels were reported (44). Another study in antibiotic-treated mice colonized with fecal microbiota from FGR patients confirmed the previous findings of altered gut microbiome in FGR patients and showed that maternal gut dysbiosis can induce placental impairment (40). The maternal gut microbiota influence on fetal-placental growth through the effects of elevated maternal *B.breve*-derived acetate was shown in mice: its interaction with maternal gut mucosa, and the effects on both placental and fetal metabolism (45). Subsequently, this translocation of bacteria or their derived metabolites from the gut to the placenta may induce changes in the placental structure and functionality under pathological conditions, and consequently, the gut-placenta axis has been proposed to play a crucial role in the etiology of PE (46, 47).

## 3 Maternal microbial metabolites translocation to the fetus

Gut microbiota can produce bioactive metabolites, including trimethylamine (TMA), trimethylamine N-oxide (TMAO), and short-chain fatty acids (SCFAs) (48). Those small molecules can enter the maternal circulation and may be actively or passively transferred throughout the placenta during fetal development (49–51). The SCFAs regulate intestinal barrier integrity and contribute to placental integrity and vascularization (52). The plasma levels of TMAO in pregnant women were found to be variable depending on the pregnancy trimester, and the levels during late pregnancy were significantly associated with a risk for developing PE (53). In addition, increased levels of LPS and TMAO, and decreased levels of SCFAs were found in plasma and feces respectively in women with PE (30, 54, 55). Since GM and its metabolites play critical roles in inflammation and hypertension, a biomarker panel consisting of intestinal bacteria and SCFAs was described as a potential tool to estimate the risk of PE (31).

Additionally, *Akkermansia muciniphila* and its metabolites (propionate and butyrate) were found to significantly ameliorate PE symptoms in a rat model by promoting autophagy and M2 polarization of macrophages in the placental bed, and propionate also promoted trophoblast invasion (31). The efficacy and safety of SCFAs in preventing insulin resistance and inflammation associated with GDM have been investigated; in this condition, metabolomic studies have been focused on metabolites derived from amino acids, carbohydrates, lipids, purines, uric acid, bile acid, and their related metabolic pathways (55–57).

The effect of a complete microbiota on the metabolite composition in placental and fetal tissues was evaluated recently using germ-free mice models, in which several compounds were found to be depleted in the fetal intestine and/or placenta (See **Supplementary Table 1**). Furthermore, the maternal microbiota strongly affects the host metabolism in the placenta and in the fetus, not only by direct production of metabolites but also by a persistent impact on host physiology (58). Regarding metabolite transport, the ABCG5 and ABCG8 transporters expressed in the placenta, liver, and intestine are known to limit the uptake of plant-derived sterols, which are themselves subject to microbiota metabolism. However, it remains unclear how far selective placental expression of any of these transporters leads to regulating fetal exposure to maternal microbial metabolites (59).

As demonstrated by gene expression and functional approaches, the solute transport proteins from the OATP and OCT families (formerly SLC21 and SLC22, respectively), and also the ABCG2 family, expressed in the placenta, are involved in the transfer of biliary products (from maternal hepatic synthesis, microbial metabolism in the maternal gut, and also from fetal synthesis) across the basal membrane in the placenta to be transformed and eliminated by the maternal liver; this transfer confers protection against high levels of bile acids in the fetoplacental unit (59, 60). Microbial constituents, such as aryl hydrocarbon ligands, induce transcriptional changes in the fetal gut, enhancing the cellularity of the innate immune system (61). Butyrate regulates the differentiation of Th17 and Th1 cells (62); maternal retinoic acid (RA) induces the generation of fetal type 3 innate lymphoid cells and, therefore, secondary lymphoid organs development (63); and *Clostridia* spp. in the maternal gut can modulate the RA levels by suppressing the expression of Rdh7 in the mouse intestinal epithelial cells (64).

The absence of a healthy microbiota is associated with deficits in immune and neuronal development, impaired stress adaptation, and metabolic dysfunction later in life, as demonstrated by several studies in germ-free animals [reviewed by (65)]. Therefore, although the existence of a placental or fetal microbiome is still controversial, and the passage of microorganisms from the mother to the fetus during *in-utero* development is uncertain, the evidence seems to suggest that at least the metabolites derived from the mother´s microbiome or pathogenic microorganisms have some effect on the fetus. Indeed, this evidence is supported by data showing how changes in the maternal fecal metabolome are reflected in the neonatal metabolome (66).

**Figure 1:**
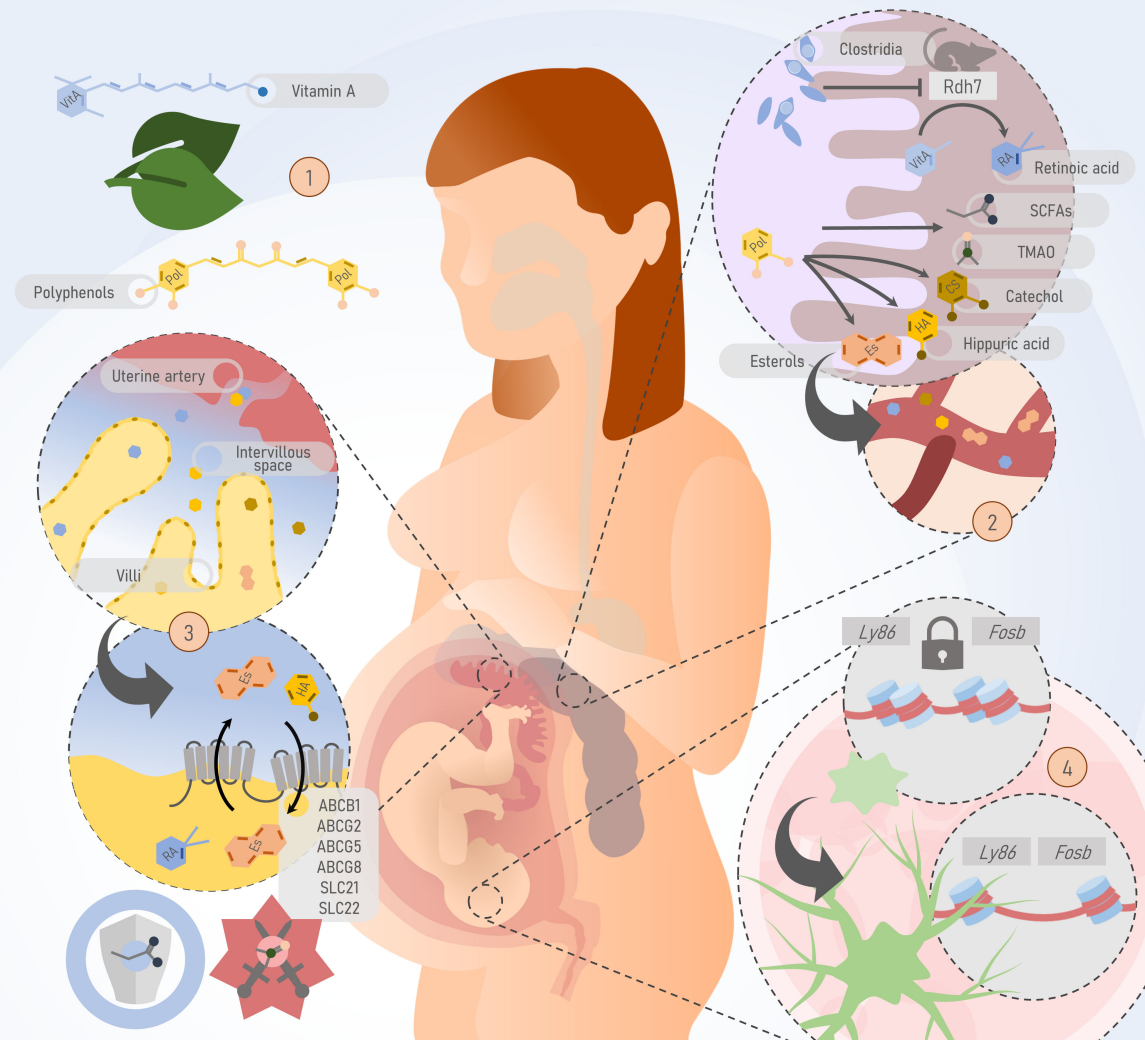
Potential pathways by which the maternal microbiota or its by-products could pass the feto-placental barrier and potentially impact fetal development and postnatal life. 1) Substrates of gut microbiome metabolism such as polyphenols and vitamin A are obtained through the diet. 2) In the gut, the maternal microbiome produces short-chain fatty acids (SCFAs), trimethylamine N-oxide (TMAO), and participates in the metabolism of polyphenols into other metabolites such as sterols, catechol, and hippuric acid. These molecules, derived from the metabolism of the gut microbiome, are passively and actively transferred to the bloodstream. In murine models, bacteria of the Clostridia class inhibit the Rdh7 enzyme, responsible for the metabolism of vitamin A in its active form, retinoic acid. 3) At the maternal-fetal interface, ATP-binding cassettes on the apical surface of the syncytiotrophoblast are responsible for the bidirectional transport of metabolites produced by the gut microbiome. SCFAs contribute to placental integrity and development, while TMAO is associated with preeclampsia. 4) Evidence from murine models shows that the absence of the maternal microbiome has a deleterious impact on processes such as microglial development through epigenetic mechanisms. Thus, microbiome-specific differentially accessible regions have been identified. For example, mouse embryos with a normal microbiome experience increased accessibility of key loci for microglial function such as Ly86 and Fosb, during development. This process does not occur in embryos from germ-free (GF) mice. *Rdh7, Retinol dehydrogenase 7; Fap2, Fibroblast Activation Protein-2; SCFAs, short chain fatty acids; TMAO, Trimethylamine N-oxide; ABC, ATP binding cassette; SLC, Solute Carrier family.*

Some of the above-mentioned metabolites, their main known effects, and their recognized transport mechanisms are illustrated in **Figure 1**.

## 4 Microbiome, metabolome, and epigenome

Epigenetics is the set of factors and molecules that modify gene expression while not implying changes in the genome sequence itself. Generally, epigenetic changes occur through modifications in DNA accessibility (i.e., DNA methylation or post-translational changes of the histones). The main interest behind epigenetics lies in the fact that, since these modifications are not directly scripted in the genetic code of the subject, even when they can be heritable material across generations, they are commonly associated with environmental cues that can make different the phenotype of two genotypically identical individuals (67). Notably, the epigenome has been proposed by some authors as one of the main factors responsible for fetal programming [reviewed by (67)], and one of the factors directly impacting the epigenome is the microbiome and its alterations.

The most apparent scenario in which microorganisms and their products can directly interact with the epigenome is in the context of an adaptive immune response. It is known that, in chronic infections, exhausted lymphocytes exhibit a unique epigenetic signature. This pattern includes decreased accessibility to memory-dependent loci such as IL-15 and IL-7R, and increased accessibility to exhaustion-related loci such as EOMES and NFATC2. More importantly, even after infection clearance, some epigenetic features persist as “scars” (68).

The capability of microorganisms to modify the host epigenome has also been confirmed beyond a pathological context and in other cell types. For example, the culture of enteroids with altered Schaedler flora, a selection of 8 dominant microorganisms in the mouse intestinal microbiome, showed that some of its components influence circadian oscillations of the epithelium through the secretion of SCFAs. These, in turn, inhibit histone deacetylases of epithelial cells, a major player in epigenetic control, ultimately leading to alterations in the expression of Bmal1 and PER2 (69).

It is known that microbiome and pathogenic microorganisms have an impact on the epigenome, and it has been proposed that modifications of the epigenome could be responsible for fetal programming; however, could it be possible that the maternal (or fetal) microbiome or infections have an impact on fetal programming through epigenetic mechanisms? In a study that followed 700 Danish children during the first six years of their life, it was described that those children who exhibited allergic rhinitis at the age of six had previously presented a microbiome of reduced diversity in the upper respiratory tract after one week of life. This finding was related to a DNA methylation pattern in mucosal cells that demonstrates a particular epigenetic signature (70). Notably, these features are accompanied by a dysfunctional response of neonatal cells to microbial products at birth, suggesting that the epigenetic characteristics of these cells are the product of *in-utero* training of the innate immune system (71). The influence of the maternal microbiome on fetal development and postnatal life has been already confirmed in experiments. In contrast to the offspring of C57BL/6J mice with a non-altered microbiome, the offspring of germ-free mice exhibit alterations in microglia development (72).

Thus, the discussion of whether the maternal microbiome and its metabolites could be involved in fetal programming by means of epigenetic changes, in the context of DOHaD, seems to favor an affirmative answer based on what has been reported in animal models.

## 5 Diseases of adulthood and their relationship to maternal microbiota

The DOHaD concept proposes that exposure to environmental factors during periods of high plasticity in the early stages of life, including the gestational period, can program the risk of the offspring developing diseases (73). The maternal microbiota is one of these factors due to its influence on the microbiome profile of the neonate (74). Just as the hygiene hypothesis raises the relevance of early exposure to microbial environments for the proper development of the immune system, the microbial hypothesis postulates the central role that this exposure plays in the composition of the microbiome, resulting in differential regulation of the immune system (75). Since the primary source of microorganisms that will colonize and shape the microbiota of the newborn comes from the maternal origin, it is worth asking whether there is a connection between dysbiosis of the maternal microbiota and the development of diseases in adulthood, such as metabolic and cardiovascular diseases, neurodevelopment alterations or changes in the immune system. **Supplementary Table 1** shows different studies describing this relationship (10).

### 5.1 Cardio-metabolic diseases

Cardio-metabolic diseases are a group of chronic noncommunicable conditions of great relevance due to their recent increase in prevalence and high mortality rate. This mainly includes cardiovascular disease, atherosclerotic disease, and diabetes, as well as associated conditions such as obesity and dyslipidemia, which may constitute risk factors for the onset of the disease (73).

The cardiovascular risk is configured by several modifiable factors, such as diet, smoking, sedentary lifestyle, and hypertension, among others (76). One of the mechanisms proposed in the study of the association between diet and cardiovascular risk is the potential of eating habits to modify the GM and consequently contribute to increased risk through changes in the metabolic pattern of these bacteria. It has been proposed that the decrease in the production of SCFAs and the increase in the secretion of TMAO are the main mechanisms involved in developing cardio-metabolic diseases (77).

Regarding the risk of developing diabetes mellitus in postnatal life, along with the role of maternal feeding during pregnancy, the antecedent of GDM is related to the presence of metabolic disorders in adulthood. One study comparing the microbiota of children from healthy mothers with children from mothers with GDM, found marked changes in microbial diversity in the healthy group (33); on the other hand, a study reported a similarity between the microbiota profiles of mothers with GDM and their children, postulating a vertical transmission of the microbiota (34).

The intestinal microbiota has also been linked to lipid metabolism. In mouse models, it has been recognized that factors such as a diet with a high intake of fatty acids and cholesterol, and the presence of dyslipidemia during pregnancy, are correlated with an increased risk of dyslipidemia and obesity in the offspring.

Multiple studies have shown a positive association between GM dysbiosis profiles and the development of obesity in adults (42, 78). It has also been reported that the microbial profiles of children are susceptible to changes due to the mode of delivery, constituting a risk factor for childhood obesity (43, 79, 80).

### 5.2 Neurodevelopment and neuropsychiatric disorders

Axonal development disruptions have been associated with maternal dysbiosis. In a murine model with dysbiosis induced by antibiotics, it has been described a decrease in levels of Netrin-G1A, and a reduction of axons at thalamocortical level, with consequent impairment in tactile and thermal sensitivity. Also, neuropsychiatric disorders such as autism have been associated with maternal dysbiosis. Multiple studies reported differences in microbial profiles among children with and without autism, and prenatal exposure to antibiotics has been postulated as a main risk factor. It has been proposed that maternal dysbiosis impacts sensitive periods of a child’s metabolic programming, which induce changes in the production of essential metabolites for neurodevelopment, as TMAO. This mechanism has been also proposed in the association between maternal obesity and anxiety disorder in the offspring (See **Supplementary Table 1**).

### 5.3 Allergy and Atopic diseases

Studies carried out with germ-free mouse models have allowed us to recognize the importance of microbial exposure offered by the GM on the process of development of the immune system in postnatal life, as well as the implication that the lack of exposure has on the increased risk for the development of allergic and autoimmune diseases (81, 82). In this model, and those with a low-diversity microbiota, increased plasma IgE levels and surface-bound IgE on mast cells were found, making these individuals more susceptible to anaphylaxis when challenged with ovalbumin injections (83).

A central change in the pathogenesis of asthma corresponds to the modification of the Th1/Th2 ratio, which a low diversity of the GM may induce (84). One study compared three germ-free mice and a control group with varying exposure to germs at different stages such as fetal, lactation, and childhood. It was evidenced that the control group, which presented a greater diversity of the intestinal microbiota, did not present clinical responses during ovalbumin inhalation, and the serum levels of IFN-γ, as well as the IFN-γ/IL-4 ratio, were significantly higher compared to the other groups. In contrast, the groups of germ-free mice showed clinical responses like asthma attacks and histological changes of alveolar inflammation.

Another study investigated the effect of administering bacterial lysates to pregnant mice on the susceptibility of offspring to develop allergic diseases, finding significant changes related to the Th1/Th2 ratio regulation, such as a higher TLR2 and TLR4 expression in the lung tissue, higher percentage of CD4+/CD35+/Foxp3+ cells in the spleen, as well as higher IL-10 concentration in plasma of the progeny of the intervention group. In contrast, the control progeny showed elevated IL-4 and IL-5 concentrations and GATA3 expression. An essential element recognized in the mothers of the intervention group was the decrease in histone deacetylase 9 expression, suggesting possible epigenetic mechanisms by which the microbiota reduces the risk of developing allergic diseases (10).

## 6 Concluding remarks

Although the passage of microorganisms from the mother to the fetus is uncertain, the evidence suggests that their derived metabolites have some effect on the fetus. Then, the influence of maternal microbiota on the offspring can be explored through the effects of their bioactive metabolites. The most studied metabolites are those byproducts of the gut microbial metabolism, such as SCFAs, TMA, and TMAO, whose variations along pregnancy are associated with complications such as PE. The microbial capability to modify the host functions is also evidenced by its interaction with the epigenome, suggesting that the maternal microbiome and its metabolites could be involved in fetal programming through epigenetic mechanisms. Then, this introduces the discussion about how dysbiosis in the maternal microbiota can be related to adulthood morbidity. The current knowledge points to the existence of a connection between dysbiosis of the maternal microbiota, and the development of diseases in adulthood, such as metabolic and cardiovascular diseases, neurodevelopment alterations, or changes in the immune system.

## Author contributions

JR-T: Conceptualization, Data curation, Investigation, Writing – original draft, Writing – review & editing. DÁ: Conceptualization, Data curation, Investigation, Writing – original draft, Writing – review & editing. AJ: Conceptualization, Data curation, Investigation, Writing – original draft, Writing – review & editing. AA: Conceptualization, Data curation, Investigation, Writing – original draft, Writing – review & editing.
