# Supplementary material for: From gut to placenta: understanding how the maternal microbiome models life-long conditions
Source: Front Endocrinol (Lausanne). 2023 Dec 15;14:1304727. doi: 10.3389/fendo.2023.1304727 (PMC10754986; doi:10.3389/fendo.2023.1304727)
Supplement: Supplementary file 1 [file Table_1.docx]

Supplementary Material

# Supplementary Table 1. Dysbiosis of the maternal microbiota and the development of diseases during pregnancy and in the offspring

|  | **Study population** | **Alterations in gut microbiota composition** | **Alterations in gut microbiota metabolites** | **Effects on offspring** | **Mechanisms of pathogenesis** | **Alterations in children´s microbiota** | **Conclusion** | **Ref.** |
| --- | --- | --- | --- | --- | --- | --- | --- | --- |
| **MATERNAL PREECLAMPSIA (PE)** | Pregnant women with PE before and after delivery were analyzed for changes in gut microbiota and compared with those of normotensive, uncomplicated pregnant women in the antepartum and 1 and 6-week postpartum periods. Antepartum (n= 48 vs 51); 1 week postpartum (n=35 vs 17); 6-week postpartum (n=18 vs 11). | 8 bacterial genera including *Blautia, Ruminococcus, Bilophila,* and *Fusobacterium* were enriched in fecal antepartum samples of PE patients compared with healthy controls while *Faecalibacterium, Gemmiger, Akkermansia, Dialister,* and *Methanobrevibacter*, were significantly depleted in this group. These antepartum alterations in gut microbiota persisted 6 weeks postpartum. | ND | ND | ND | ND | Early-onset PE is associated with significant gut microbial alterations in antepartum and postpartum women. | (28) |
|  | Systematic review of six studies with an overall sample size of 416 PE women and 704 healthy controls. | PE patients had elevated Bacteroidetes and Proteobacteria, and depleted Firmicutes and Verrucomicrobia compared to healthy women. *Fusobacterium* and *Ruminococcus* were enriched and *Lachnospira, Akkermansia, Faecalibacterium, Bifidobacterium* and *Alistipes* were depleted in PE. | ND | ND | ND | ND | There is a similar dysbiosis in the intestinal microbiota of patients with PE compared to healthy women. | (29) |
|  | 48 PE patients and 48 healthy pregnant women. | At the phylum level, the abundances of Bacteroidetes, Proteobacteria, and Actinobacteria were higher, while the abundance of Firmicutes was lower in patients with PE. | Increased levels of plasma TMAO in patients with PE compared to healthy controls. | ND | PE patients had a gut microbiota dysbiosis and increased plasma LPS and TMAO levels, which were related to inflammation. | ND | Patients with PE have significant dysbiosis of the gut microbiota, as well as increased levels of plasma LPS and TMAO, compared to healthy controls. | (30) |
|  | 92 PEs and 86 normal late-pregnant women. | Decrease of SCFA-producing bacteria in feces (including *Akkermansia muciniphilus*). | Decrease of fecal, serum, and placental levels of SCFAs (propionic acid and butyric acid). | ND | Increased inflammation and impaired intestinal barrier function: decrease of peripheral Tregs, Treg/Th17 cell ratios; serum levels of IL-10 and fecal levels of 2AG. Increase of Th17 cells and serum levels of IL-17, IL-1β, and LPS. | ND | Gut dysbiosis plays a significant role in the development of PE. | (31) |
|  | 20 PE patients and 26 normotensive pregnant women. | Enriched Bacteroidetes and depleted Verrucomicrobia and Syntergistota at the phylum level and depleted *Akkermansia* at the genus level in the feces of PE patients. | ND | ND | ND | ND | There are significant differences in the composition of the gut microbiota between pregnant women with preeclampsia and those with normotensive pregnancies. | (32) |
| **GESTATIONAL DIABETES MELLITUS (GDM)** | 34 newborn meconium samples of mothers diagnosed with GDM (n=20) and unaffected mothers (n=14). | ND | ND | The newborn weight and height at birth did not differ significantly among GMD groups and controls. No other effects were evaluated. | ND | Absence of different phyla such as Proteobacteria, Firmicutes, Actinobacteria, Bacteroidetes, Chloroflexi, Acidobacteria, and Planctomycetes. Increase in Actinobacteria abundance as well as decrease in Bacteroidetes levels. | Marked reduction in bacterial diversity in meconium samples from neonates born to mothers diagnosed with GDM. | (33) |
|  | 83 Infant meconium samples from GDM and non-GDM mothers. | Alterations in the oral, intestinal and vaginal microbiota of pregnant women with GDM compared to healthy women. Some of the genera that showed significant differences included *Porphyromonas, Bacteroides, Blautia, Coprococcus, Roseburia, and Sutterella.* | The study found that GDM was associated with lower evenness of Kegg orthologues, indicating a decline in metabolic capacities in the meconium microbiota of neonates of GDM mothers. | GDM has been associated with an increased risk of fetal macrosomia, respiratory distress syndrome, and type 2 diabetes in the offspring. | Transmission of altered maternal microbiota to the newborn during pregnancy or delivery, and the increased fetal exposure to glucose, may increase the risk of complications and have long-term consequences for the offspring. | There were differences in the abundance of certain genera, such as *Prevotella, Streptococcus, Bacteroides, and Lactobacillus*, in the neonatal microbiota associated with GDM. | GDM can lead to significant alterations in the maternal and neonatal microbiota. | (34) |
|  | 46 Mother-infant (13 with GDM and 33 normal pregnant women). | ND | Decrease levels of certain SCFAs levels were observed in infants born to mothers with GDM. | Alterations in the microbiota composition of offspring born to mothers with GDM. | Transmission of altered maternal gut microbiota to the infant during pregnancy and childbirth, and transmission of inflammatory mediators from the mother to the fetus. | Alterations in the abundance of microbial taxa, such as decreased levels of *Lactobacillus, Flavonifractor, Lactobacillaceae, Rikenellaceae, Erysipelotrichaceae,* and certain families within *Gammaproteobacteria*. | GDM is associated with alterations in the early colonization of the infant gut microbiota, as well as changes in the levels of SCFAs in the infant gut. | (35) |
|  | 418 Mother-infant (147 with GDM and 271 normal pregnant women). | ND | Certain metabolites, including GPC, glycocholic acid, rhamnose, riboflavin, and taurine, showed changes in their abundances in both the meconium and maternal blood of the GDM group. | Neonates born to mothers with GDM showed alterations in their meconium microbiota and metabolome. | Translocation of maternal gut microbiota to the fetus during pregnancy, which can influence the colonization and development of the infant gut microbiota. | An increase in alpha diversity, a decrease in Proteobacteria, and an increase in Firmicutes was reported. | GDM is associated with alterations in the composition of the meconium microbiota and metabolome in neonates. | (36) |
|  | 84 Obese mother-  infants (40 with GDM and 44 normal pregnant women). | ND | ND | ND | ND | No significant differences in the overall composition of the gut microbiota were found between the offspring of mothers with and without GDM. | Previous gestational diabetes mellitus (GDM) status did not significantly affect the overall composition of the gut microbiota in postpartum mothers and their offspring. | (37) |
|  | 120 Mother-infants (60 with GDM and 60 normal pregnant women). | ND | ND | Offspring born to mothers with GDM had higher BMI Z-scores compared to offspring of healthy controls. | Transmission of altered maternal gut microbiota to the offspring. The altered microbiota composition and function can influence nutrient absorption, energy balance, and inflammation, potentially contributing to the development of obesity and metabolic disorders in the offspring. | It reports a reduction in alpha and beta diversity. The abundance of certain genera, such as *Burkholderia, Caballeronia, Paraburkholderia, Aerococcus,* and *Faecalibacterium* was found to be significantly lower in the meconium microbiota of infants born to mothers with GDM. The abundance of genera like *Xanthobacter, Cytophaga, Serratia,* and *Actinomyces* was significantly higher in this group. | GDM is associated with alterations in the meconium microbiota of offspring, these changes in the microbiota may contribute to increased infant BMI. | (38) |
| **FETAL GROWTH RESTRICTION (FGR)** | Fecal samples from 14 pregnant women with FGR and 18 normal controls. | *Bacteroides, Faecalibacterium,* and *Lachnospira* were highly abundant in the FGR subjects. | ND | ND | ND | ND | These results indicated a relationship between maternal dysbiosis during pregnancy and the risk of FGR, which might involve the dysregulation of glycometabolism. | (39) |
|  | 35 women with FGR and 35 with normal pregnancies. | Relative abundances of *Lactobacillus* and *Catenibacterium* were elevated in the FGR group, while the abundances of *Ruminococcaceae, Bacteroides uniformis*, *Mollicutes* RF39, and *Alistipes onderdonkii* decreased. | Alterations in metabolic pathways such as nicotinate and nicotinamide; butanoate; histidine; and alanine, aspartate, and glutamate metabolism. | The FGR group showed significantly decreased fetal weight and crown-rump length. | ND | ND | Dysbiosis of the gut microbiota in FGR patients may contribute to placental insufficiency and fetal malnutrition. | (40) |
|  | 8 women with FGR and 8 with normal pregnancies. | There was no significant difference in the gut microbiota composition between FGR and normal pregnant women but a total of 20 gut microbes were significantly different between two groups, and the correlation analysis found that *Roseomonas* and *Propionibacteriaceae* were significantly positively correlated with both maternal body mass index (BMI) before delivery, placental weight, and neonatal birth weight (BW) percentile, while *Marinisporobacter* and *Sphingomonas* were significantly negative correlated with both neonatal BMI and neonatal BW percentile. | ND | ND | ND | ND | The occurrence of FGR was closely associated with the differential gut microbes of pregnant women in their third trimester of pregnancy. *Propionibacteriaceae* was a beneficial bacterium, while *Marinisporobacter* and *Sphingomonas* were harmful. | (41) |
| **MATERNAL OBESITY** | 256 women with different BMI | *Bacteroides* group, Clostridium group, and Staphylococcus were increased in overweight women compared to normal-weight women. | ND | Children's intestinal microbiota composition was correlated with the maternal BMI. Additionally, maternal obesity was associated with higher birth weight and an increased risk of obesity during adolescence. | Specific microbial compositions, such as the *Bacteroides* group and Staphylococcus, have been found to be altered in overweight individuals, and high concentrations of these microorganisms may be associated with enhanced energy storage and obesity, as well as increased inflammation. | * High BMI mothers: a lower abundance of species such as *Bifidobacterium* and higher abundance of *Bacteroides* and *Staphylococcus*.  * Low BMI mothers: a lower abundance of *Akkermansia muciniphila*, *Staphylococcus,* and *Clostridium difficile.* | Maternal BMI, weight, and weight gain during pregnancy influence the composition and development of infant GM. | (42) |
|  | 935 full-term infants with different modes of delivery, from obese and normal-weight women. | Women with a high pre-pregnancy body mass index (BMI) have elevated levels of *Bacteroides* in their third trimester compared to normal-weight women. | ND | * Composition of the infant gut microbiota was influenced by both maternal weight status and birth mode:  Infants from obese mothers born vaginally had 3 times more risk of developing obesity than infants from normal-weight mothers.  * Infants born by cesarean section had 5 times more risk than those whose mothers had adequate weight. | Transfer of obesogenic microbes from the mother to the newborn. This transfer can occur during pregnancy, birth, and early infancy, and it is hypothesized that these microbes can influence energy metabolism, adipogenesis, and inflammation. | Among overweight or obese children (OWOB) born to OWOB mothers, the family Lachnospiraceae became more abundant with increasing levels of several other families, including Lactobacillales, Ruminococcaceae, and Veillonellaceae.  Few of the same interactions for the family Lachnospiraceae were seen among OWOB children after normal-weight pregnancy. | Both maternal weight status and cesarean delivery shape early-life gut microbial development and the weight outcome of offspring. | (43) |
| **CARDIOMETABOLIC DISEASES** | Animal model: The male offspring of dams fed on either a high-fat (HF) diet or control (C) diet and then weaned to either an HF or C diet, generating four groups: C-C, HF-C, C-HF and HF-HF. | ND | ND | The male offspring of the C-HF and HF-HF groups had higher body weight, glycemia levels, and insulin resistance than the C-C group. | Changes in the gut microbiota could be due to the interaction between maternal and post-weaning diet, which predisposes the offspring to aberrant glucose metabolism and alterations of gut microbiota in later life. | The C-HF offspring compared with HF-C and C-C, showed a significantly lower diversity. The Verrucomicrobias were significantly increased and Bacteroidetes were significantly decreased in C-HF and HF-HF groups, compared with C-C and HF-C groups. | Maternal and post-weaning diet interaction predisposes the offspring to aberrant glucose metabolism and alterations of gut microbiota in later life | (76) |
|  | Animal model: Wistar rats fed with high-fat and high-cholesterol (HFHC) diet-induced maternal dyslipidemia. The probiotic *L. plantarum* WJL during pregnancy and lactation was administered. | The consumption of an HFHC diet increased the relative abundance of Firmicutes and Fusobacteria phyla, while Bacteroidetes, Verrucomicrobia, and Actinobacteria phyla were reduced. | The consumption of an HFHC diet during gestation and lactation led to a decrease in the levels of SCFAs, including acetate, propionate, and butyrate by gut microbiota. | Higher LDL cholesterol levels and a significant reduction in HDL cholesterol levels compared to the offspring of control mice. Additionally, it was found that increased blood pressure, endothelial dysfunction, and a higher risk of developing cardiovascular diseases than offspring of control mice. | Diets with high-fat content could reduce the alpha and beta diversity of the intestinal microbiota, causing dysbiosis. Other mechanisms are the effects of dyslipidemia as inflammation, epigenetic modifications, and metabolic disturbances, such as reduction in SCFA production. | ND | Administration of the probiotic *L. plantarum* WJL during pregnancy and lactation in dams improved gut microbiota diversity reduced maternal dyslipidemia and prevented cardiovascular dysfunction in male rat offspring. | (77) |
|  | Animal model: mice fed with a High-fructose (HF) diet during pregnancy and lactation. | ND | HF-diet induced an increase in plasma acetate level.   On the other hand, administration of probiotics or prebiotics induced the production of propionate. | It was found that the group receiving HF- diet had the highest blood pressure values.   Groups receiving probiotics or prebiotics had a decrease in blood pressure values compared to the group receiving only an HF-diet. | Acetate is a ligand for the Olfr78 receptor at the afferent arteriola level, which stimulates the activation of the renin-angiotensin-aldosterone system, so its effect is hypertensive.   Propionate acts as a ligand of the GPR41 receptor, whose effect is vasodilatation, and therefore a hypotensive effect. | *HF-diet led to an increase in the abundance of the genus *Akkermansia* and a decrease in the abundance of the genera *Bacteroides* and Prevotella  *Groups that received probiotics or prebiotics presented a reduction in the Actinobacteria/firmicutes ratio, which has been proposed as a microbial marker of arterial hypertension. | HF-diet during pregnancy and lactation leads to the development of hypertension in adult male offspring. This is associated with alterations in the gut microbiota composition. | (78) |
|  | Animal model: the offspring of Wistar rats fed a dyslipidemic diet received a saline solution or the probiotic *L. fermentum* for 8 weeks. | ND | ND | Male offspring that received probiotic formulation exhibited less body weight, reduced plasma triglycerides levels, increased HDL levels, and reduced mean arterial pressure compared to those that did not. | Dyslipidemia can lead to oxidative stress, inflammation, and alterations in the gut microbiota composition. | The intervention with probiotic formulation increased fecal counts of *Lactobacillus spp*. and decreased fecal counts of *Enterobacteriaceae* when compared to those that did not receive the intervention. | The probiotic formulation containing *L. fermentum* improved lipid profile and blood pressure values in male offspring exposed to maternal dyslipidemia. | (79) |
|  | Animal model: mice fed with a high-fat diet (HFD) or Normal-chow diet (NCD). | The HFD group had lower microbial richness and diversity compared to the NCD group. | ND | The offspring of HFD-fed mothers showed impaired glucose tolerance, insulin sensitivity, and insulin secretion compared to the offspring of mothers on an NCD. | Certain microbial strains present in the maternal groups were transmitted to the offspring, indicating vertical transmission of the maternal core gut microbiota. | Increase in the relative abundance of *Blautia, Romboutsia,* and *Muribaculaceae.*   The study found differential production of metabolites in the offspring of mice fed with HFD such as dipeptides containing branched-chain amino acids, as well as salicylic acid and isobutyric acid. | Correlation between maternal and offspring profiles and metabolic affectations was evidenced. | (80) |
|  | Animal model: Wistar rats with hypertension that received captopril during pregnancy and lactation. | Maternal treatment with captopril altered microbial composition: genus, *Allobaculum,* family *Erysipelotrichaceae*, and the class *Erysipelotrichia* were enriched; the order *Clostridiales* and class Clostridia were more abundant (genera considered beneficial in the order Clostridiales showed high average abundance). | ND | * Maternal hypertension resulted in the development of hypertension in adult male offspring  * Maternal captopril treatment improved gut inflammation and permeability in male offspring of hypertensive rats; it also ameliorated autonomic neuroinflammation. | Maternal arterial hypertension is associated with dysregulation of the gut-brain axis in the offspring, and the activation of the renin-angiotensin aldosterone system. | Bacterial phylotypes enriched in the offspring of mothers treated with captopril were multiple bacterial genera in the *Clostridiales Order: Anaerostipes, Coprococcus, Oscillospira, Roseburia, Dehalobacterium.* | Maternal captopril treatment persistently alters the gut-brain axis and attenuates hypertension of male offspring; this effect may be partially mediated by modulation of the gut microbiota by captopril. | (81) |
| **NEURODEVELOPMENT AND NEUROPSYCHIATRIC DISORDERS** | Animal model: Female mice received gut microbiota transplants from mice fed high- or low-fat diets (HFD or LFD). Recipient mice were then bred. | ND | ND | Male offspring fed HFD showed a more anxious profile associated with more agitated and compulsive behaviors. | Certain metabolites produced by gut bacteria, such as SCFAs and tryptophan metabolites, have been implicated in brain function. Disruption of the maternal gut microbiota due to an HFD can lead to dysbiosis and dysfunction in the production of these metabolites. | ND | HFD-induced maternal dysbiosis is sufficient to disrupt behavioral function in murine offspring in a sex-specific manner. | (82) |
|  | Animal model: Murine embryos of mothers treated with antibiotics; embryos of germ-free mice, and finally, embryos of mothers treated with antibiotics that were recolonized, and a control group. | ND | ND | Impaired tactile sensitivity in the hind and fore legs and impaired thermal sensitivity were reported in antibiotic-treated and germ-free mice, which was not evident in recolonized mice. | In the first two groups, it was possible to evidence a decrease in the embryo levels of the protein Netrin-G1A and in the expression levels of the coding gene. | ND | Depletion of the maternal microbiota, either through antibiotic treatment or germ-free upbringing, resulted in abnormalities in fetal brain gene expression and impaired axonogenesis. | (83) |
|  | Mothers exposed to antibiotic therapy during gestation. | It was reported that prenatal antibiotic exposure was associated with alterations in the composition of the maternal microbiota, but specific alterations were not mentioned. | Prenatal antibiotic exposure has been shown to induce changes in metabolites such as TMAO, trimethyl-5-amino valerate, imidazole propionate, 3-indole sulfate, and hippurate. | Prenatal antibiotic exposure was associated with an HR of 1.1 (95% CI, 1.05- 1.15) for developing autism spectrum disorder, especially in the first and second semesters. | The dysbiotic microbiome and the altered metabolites produced by it may disrupt the communication between the gut and the brain. Animal studies have shown that these metabolites, such as trimethylamine oxide (TMAO), imidazole propionate, 3-indole sulfate, and Hippurate, can influence brain function and neuronal communication. | ND | Prenatal antibiotic exposure had a slight increase in the risk of autism spectrum disorder (ASD) in offspring. However, when analyzing discordant sibling pairs, the association was attenuated, suggesting that unmeasured genetic and environmental factors may influence the association. | (84) |
| **ALLERGY AND ATOPIC DISEASES** | Animal model: three groups of germ-free mice and a control group, which had different exposures to germs at different periods such as fetal, lactation, and childhood. | ND | ND | Germ-free mice developed a clinical response like an asthma attack; histological analysis showed a widening of the alveolar septa, marked cellular and inflammatory infiltrate, and capillary dilatation. | ND | The diversity of intestinal microflora of offspring kept in a general environment during all periods was significantly higher than in other groups. | The composition of the maternal microbiota can influence the development of the offspring's microbiota and immune system. | (85) |
|  | 12,688 mother-child pairs (from Danish National Birth Cohort), were exposed to antibiotic therapy during gestation. | ND | ND | Prenatal exposure to antibiotics was associated with an increased risk of atopic dermatitis in children born to mothers with atopy, but only when antibiotics were used in both the 1st-2nd and 3rd trimester of pregnancy. | Antibiotics can disrupt the maternal microbiota during pregnancy, leading to alterations in the composition and diversity of the microbiota. This disruption can potentially affect the development and maturation of the immune system in the offspring. | ND | Prenatal exposure to antibiotics is associated with an increased risk of atopic dermatitis in children born to mothers with atopy. | (86) |
|  | Animal model: pregnant mice administering bacterial lysates. | Oral administration of bacterial lysates during pregnancy resulted in changes in the composition of the maternal intestinal microbiome, with enrichment of Firmicutes and Proteobacteria phyla and changes in specific bacterial families such as *Ruminococcaceae* and *Helicobacteraceae.* | ND | *Control group:  presence of pathological changes of asthma in the pulmonary histological analysis, such as higher cell counts with predominance of eosinophils and high levels of IgE in plasma.  * Intervention group: expression of TLR2 and 4 in lung tissue, higher percentage of Treg cells in the spleen, as well as higher IL-10 concentration in plasma. | Th1/Th2 ratio regulation. | The mice that received the bacterial lysates presented greater bacterial richness in fecal samples, with a high abundance of Firmicutes and Proteobacteria, compared to controls. | Oral administration of bacterial lysates during pregnancy can modulate maternal intestinal microbiota and have beneficial effects on immune regulation. | (10) |

To illustrate the connection between dysbiosis of the maternal microbiota and the development of diseases during pregnancy and in the offspring's adulthood, we summarize in this table the alterations in gut microbiota composition observed in pregnancy complications, such as PE, GDM, and FGR by several studies. In addition, those maternal alterations, and the alterations in offspring´s microbiota are associated with the development of diseases in the children, such as metabolic and cardiovascular diseases, neurodevelopment alterations, or changes in the immune system from different experimental models. *2AG: 2-Arachidonoylglycerol; BMI: Body mass index; GPC: glycerophosphocholine; LPs: normal late-pregnant women; LPS: lipopolysaccharide; ND: not described; SCFAs: short chain fatty acids; TMAO: trimethylamine-N-oxide.*
